# Xinmailong Attenuates Doxorubicin-Induced Lysosomal Dysfunction and Oxidative Stress in H9c2 Cells via HO-1

**DOI:** 10.1155/2021/5896931

**Published:** 2021-03-27

**Authors:** Yu Jiang, Yanjuan Liu, Wen Xiao, Dandan Zhang, Xiehong Liu, Huiqiong Xiao, Sanli You, Lili Yuan

**Affiliations:** ^1^Hunan Provincial Key Laboratory of Emergency and Critical Care Metabonomics, Hunan Provincial Institute of Emergency Medicine, Hunan Provincial People's Hospital/The First Affiliated Hospital of Hunan Normal University, Changsha, Hunan, China; ^2^Department of Cardiology, Brain Hospital of Hunan Province, Changsha, Hunan, China

## Abstract

The clinical use of doxorubicin (DOX) is limited by its cardiotoxicity, which is closely associated with oxidative stress. Xinmailong (XML) is a bioactive peptide extracted from American cockroaches, which has been mainly applied to treat chronic heart failure in China. Our previous study showed that XML attenuates DOX-induced oxidative stress. However, the mechanism of XML in DOX-induced cardiotoxicity remains unclear. Heme oxygenase-1 (HO-1), an enzyme that is ubiquitously expressed in all cell types, has been found to take antioxidant effects in many cardiovascular diseases, and its expression is protectively upregulated under DOX treatment. Lysosome and autophagy are closely involved in oxidative stress as well. It is still unknown whether XML could attenuate doxorubicin-induced lysosomal dysfunction and oxidative stress in H9c2 cells via HO-1. Thus, this study was aimed at investigating the involvement of HO-1-mediated lysosomal function and autophagy flux in DOX-induced oxidative stress and cardiotoxicity in H9c2 cells. Our results showed that XML treatment markedly increased cell proliferation and SOD activity, improved lysosomal function, and ameliorated autophagy flux block in DOX-treated H9c2 cells. Furthermore, XML significantly increased HO-1 expression following DOX treatment. Importantly, HO-1-specific inhibitor (Znpp) or HO-1 siRNA could significantly attenuate the protective effects of XML against DOX-induced cell injury, oxidative stress, lysosomal dysfunction, and autophagy flux block. These results suggest that XML protects against DOX-induced cardiotoxicity through HO-1-mediated recovery of lysosomal function and autophagy flux and decreases oxidative stress, providing a novel mechanism responsible for the protection of XML against DOX-induced cardiomyopathy.

## 1. Introduction

Doxorubicin (DOX), a broad-spectrum anthracycline anticancer drug, is widely used in the chemotherapy of various tumors [1]. However, its clinical application is limited by dose-dependent cardiotoxicity [2]. DOX-induced cardiotoxicity is caused by multifactors. Nevertheless, the precise molecular mechanisms of epirubicin-induced cardiotoxicity remain elusive. Oxidative stress is considered an important cause of DOX-induced cardiotoxicity, which has been the prevailing hypothesis [3]. Lysosome and autophagy are closely involved in oxidative stress as well. The lysosome is responsible for cellular homeostasis through digesting unwanted materials in the cells [4]. Autophagy, regarded generally as a protective mechanism that maintains cell proliferation, is a vital cellular process of protein and organelle recycling [5], while it is nevertheless subject to dysregulation having detrimental effects on the cell. Some articles have shown that DOX induces cellular changes consistent with autophagy initiation and autophagosome formation in cardiac cells. Autophagic cell death has been proposed to contribute to DOX-induced cardiotoxicity. The fusion of the lysosome and autophagosome to form an autolysosome is a critical step during the process of autophagy, and accumulation of autolysosomes leads to increased reactive oxygen species (ROS) production [6]. Numerous endogenous or exogenous antioxidants can alleviate DOX-induced cardiotoxicity [7, 8]. However, specific targets and mechanisms of oxidative stress remain to be identified.

Xinmailong (XML) is composed of complex nucleobase and binding amino acids, which are extracted from American cockroaches [9] and have been mainly used to treat chronic heart failure (CHF) in China [10]. Meta-analysis shows that XML, together with conventional treatment, may improve cardiac function in patients with CHF with few adverse effects [11]. XML shows effective protection against cardiovascular injury, including DOX-induced cardiotoxicity [12]. Our previous study showed that XML attenuates DOX-induced oxidative stress [13]. However, its mechanism is still not clear.

Heme oxygenase-1 (HO-1), an enzyme that is ubiquitously expressed in all cell types, catalyzes the degradation of heme, and its induction and expression are critical protective mechanisms during cell oxidative stress [14]. HO-1 takes antioxidant effects in various cardiovascular diseases, and its expression is protectively upregulated under DOX treatment [15]. Considering that both XML and HO-1 have antioxidant effects, our study evaluated the hypothesis that XML attenuates doxorubicin-induced lysosomal dysfunction and oxidative stress in H9c2 cardiomyocytes via HO-1. To verify this hypothesis, we treated the DOX-induced H9c2 cell model with XML and inhibited HO-1 expression and observed lysosomal function and oxidative stress. We demonstrate that XML attenuates DOX-induced lysosomal dysfunction and oxidative stress and upregulates HO-1 expression. HO-1 deficiency inhibits the protective role of XML. These results indicate that XML acts as an effective treatment for DOX-induced H9c2 cardiotoxicity and its underlying molecular mechanism is identified.

## 2. Materials and Methods

### 2.1. Cell Culture

H9c2 cells were derived from the rat embryonic heart (ATCC, CRL-1446). H9c2 cells were cultured in Dulbecco's Modified Eagle's Medium (DMEM) (Gibco, Grand Island, NY, USA, cat: 11965092), supplemented with 10% fetal bovine serum (Gibco, Grand Island, NY, USA, cat: 10100139C) and 1% penicillin-streptomycin (10.000 U penicillin and 10 mg streptomycin/mL, Gibco), and maintained at 37°C and 5% CO_2_. H9c2 cells were maintained in the exponential growth phase and passaged when they reached about 80% confluence at a split ratio of 1 :  3. Firstly, remove and discard the culture medium and wash H9c2 cells 3 times by adding 4-5 mL PBS (phosphate-buffered solution) to remove all traces of serum that contains a trypsin inhibitor. Then, add about 1 mL of trypsin-EDTA solution and observe cells under an inverted microscope until the cell layer is dispersed (usually within 1 to 2 minutes). Add 4-5 mL of complete medium culture and aspirate cells by gently pipetting. Lastly, aspirate appropriate aliquots of the cell suspension to new culture vessels and incubate the cultures at 37°C in 5% CO_2_.

### 2.2. siRNA Transfection

HO-1 siRNA (5′-GGAAAAUCCCAGAUCAGCATT-3′) was synthesized by RiboBio (China). The siRNA was transfected into H9c2 cells for 48 h using riboFECT™ CP (RiboBio, cat: C10511-05) according to the manufacturer's instructions.

### 2.3. Establishment of DOX Cardiotoxicity

H9c2 cells were treated with 5 *μ*mol/L DOX (Sigma, cat: D1515) for 24 h to establish the DOX cardiotoxicity cell model. H9c2 cells were preincubated with 0.25, 0.5, or 1 mg/mL XML (Yunnan Tengyao, cat: Z20060443) for 48 h before model establishment, and the HO-1 inhibitor 5 *μ*mol/L Znpp (MedChemExpress, cat no. HY-101193) or HO-1 siRNA was used to interfere with HO-1 for 48 h before model establishment.

### 2.4. Measurement of H9c2 Cell Proliferation

The Cell Counting Kit-8 (CCK-8) assay was used to screen the cytotoxic or protective activity of XML in the DOX cardiotoxicity cell model. The cell proliferation [16] was determined with CCK-8 (Dojindo, cat: CK04) according to the manufacturer's instructions. H9c2 cells were seeded into 96-well culture plates at a density of 2 × 10^3^ cells per well and grown for 24 h. After the corresponding treatment, 10 *μ*L CCK-8 solution was added to each well of the plate, and then, the plate was incubated for 1 hour in the incubator. Finally, the absorbance at 450 nm was measured using a microplate reader ELx808 (BioTek). The optical density value was converted to the relative cell proliferation rate to the control group.

### 2.5. Measurement of H9c2 Oxidative Stress

Cell oxidative stress [17] was measured by using superoxide dismutase (SOD), and a total SOD activity assay utilizes a tetrazolium salt WST-8 that produces a water-soluble formazan dye upon reduction with superoxide anion. H9c2 cells were incubated in 60 mm dish plates at a density of 4 × 10^5^ cells overnight. After the corresponding treatment, cells were collected and measured with a total SOD activity kit (Beyotime, cat: S0101) according to the manufacturer's instructions.

### 2.6. Measurement of Lysosome

Lysosome function [18] was determined by using LysoTracker Red (Beyotime, cat: C1046), which was a fluorescent acidotropic probe for labeling and tracking acidic organelle lysosomes. LysoTracker Red probes, consisting of a fluorophore linked to a weak base, are freely permeant to cell membranes and typically concentrate in spherical organelles, thus forming the LysoTracker Red puncta. Firstly, H9c2 cells were incubated in 60 mm dish plates at a density of 4 × 10^5^ cells overnight. After the corresponding treatment, cells were incubated with 50 nM LysoTracker Red for 1 h and then imaged with a fluorescence microscope (Olympus). LysoTracker Red was excited with a 577 nm laser and detected with a 590 nm filter. LysoTracker Red puncta were examined microscopically at ×200 magnification.

### 2.7. Measurement of Lysosomal pH

Lysosomal pH [19] was determined by using fluorescein-dextran (Sigma, cat: FD40). H9c2 cells were incubated in 96-well plates at a density of 2 × 10^3^ cells overnight. After the corresponding treatment, 0.2 mg/mL fluorescein-dextran was added to each well and incubated for 1 h. The cells were washed three times with PBS and incubated for 2 h. The 490 and 440 nm fluorescence values were measured, and the 490/440 ratio was calculated and conversed into a pH value.

### 2.8. Real-Time PCR Analysis

Real-time PCR was used to analyze mRNA expression. H9c2 cells were incubated in 60 mm dish plates at a density of 4 × 10^5^ cells overnight. After the corresponding treatment, cells were harvested and subjected to real-time PCR analysis. Briefly, total RNA was extracted by using TRIzol (Invitrogen, cat: 15596-026) according to the manufacturer's instructions. Subsequently, cDNAs were synthesized by using PrimeScript™ RT Reagent Kit (Takara, cat: RR037). HO-1 mRNA expression was measured by using TB Green® Premix Ex Taq™ (Takara, cat: RR420) using StepOnePlus (Applied Biosystems). The primers were as follows: for HO-1, sense 5′-GAAACCAGCAGCCCCAAATC-3′ and antisense 5′-ATCAAAGTGGCCATGACGCT-3′; for GAPDH, sense 5′-AGTGCCAGCCTCGTCTCATA-3′ and antisense 5′-GACTGTGCCGTTGAACTTGC-3′; and for *β*-actin, sense 5′-CCCATCTATGAGGGTTACGC-3′ and antisense 5′-TTTAATGTCACGCACGATTTC-3′. The thermal cycling parameters were as follows: predenaturation at 95°C for 30 sec, cycling stage at 95°C for 15 s, 60°C for 34 s, and 40 cycles. The 2^−ΔΔCt^ method was used to analyze the relative gene expression data. The mRNA levels of HO-1 were normalized to those of GAPDH and *β*-actin.

### 2.9. Western Blot Analysis

Protein expressions were semiquantified by Western blotting [20]. H9c2 cells were incubated in 60 mm dish plates at a density of 4 × 10^5^ cells overnight. After the corresponding treatment, cells were harvested and subjected to Western blotting analysis. Briefly, total protein was extracted by using RIPA lysis buffer (Beyotime, cat: P0013B) and quantified by using a BCA kit (Beyotime, cat: P0012S). Subsequently, 20 *μ*g of proteins was separated using 12% SDS-PAGE and transferred to the PVDF membrane. The membranes were blocked in 5% defatted milk and incubated with primary antibodies at 4°C overnight, LC3 (L7543, dilution 1 : 3000, rabbit, Sigma-Aldrich), p62 (ab91526, dilution 1 : 3000, rabbit, Abcam), *β*-actin (ab8227, dilution 1 : 3000, rabbit, Abcam), and HO-1 (ab13248, dilution 1 : 3000, rabbit, Abcam), and then incubated with a goat anti-rabbit secondary antibody (ab6721, dilution 1 : 10000, goat, Abcam) for 1 h. Finally, the membrane was incubated with an enhanced chemiluminescence (ECL) substrate reagent (Thermo Pierce, cat: 32106) and recorded using ImageQuant LAS 500 (GE). The intensities of the protein bands were measured using ImageJ software ver. 1.8 (National Institutes of Health).

### 2.10. Immunofluorescence

Double immunofluorescence [21] was used to detect LC3 and p62 protein expressions as well. H9c2 cells were incubated on the cover glass which was placed in 60 mm dish plates at a density of 4 × 10^5^ cells overnight. After the corresponding treatment, the cells were fixed with 4% paraformaldehyde and handled with 1% Triton X-100 for 20 minutes. Then, the cells were blocked in PBS with 10% fetal bovine serum for 30 min. The fixed cells were incubated with the primary antibody of LC3 (L7543, dilution 1 : 200, rabbit, Sigma) and p62 (ab91526, dilution 1 : 3000, rabbit, Abcam), followed by incubation with a fluorescent secondary antibody (ab6721, dilution 1 : 200, goat, Abcam). Cells were washed with PBS three times, and nuclei were stained with DAPI at a dilution of 1 : 500 (Beyotime, cat: C1002) in the dark for 1 h and imaged using a fluorescence microscope IX51 (Olympus) under the excitation wavelength of 532 nm and the emission wavelength of 588 nm for LC3 (red), under the excitation wavelength of 488 nm and the emission wavelength of 518 nm for p62 (green), and under the excitation wavelength of 340 nm and the emission wavelength of 488 nm for DAPI (blue).

### 2.11. Statistical Analysis

All data are shown as the means ± standard deviation (SD). Data analysis and figure generation were performed with GraphPad Prism 7.0 (GraphPad Software). Data are representative of at least three independent experiments. Significant differences among the groups were identified by the independent one-way ANOVA and the Tukey-Kramer test. *P* < 0.05 was considered statistically significant.

## 3. Results

### 3.1. Effect of XML on Oxidative Stress in DOX-Induced H9c2 Cells

**Figure 1:**
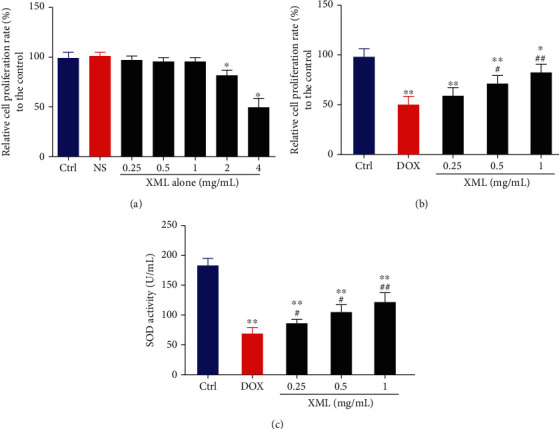
XML alleviates the increased cell proliferation and SOD activity in DOX-induced H9c2 cells. (a, b) Cell proliferation was assessed by CCK-8. (c) Cell oxidative stress was reflected by SOD activity. H9c2 cells were treated with 0.25, 0.5, 1, 2, and 4 mg/mL XML alone for 24 h. H9c2 cells were treated with DOX for 24 h only or preincubated with 0.25, 0.5, and 1 mg/mL XML for 48 h. Data are representative of at least three independent experiments (mean ± SD). Statistical significance was assigned as ^∗^*P* < 0.05 and ^∗∗^*P* < 0.01 compared with control and ^#^*P* < 0.05 compared with DOX. *P* values were determined using the independent one-way ANOVA and the Tukey-Kramer test. XML: Xinmailong treatment; DOX: doxorubicin; CCK8: Cell Counting Kit-8; SOD: superoxide dismutase.

To determine the dose response of XML in H9c2 cells, we evaluated the proliferation of H9C2 cells. As shown in Figure 1(a), cell proliferation was not influenced by 0.25 to 1 mg/mL XML, while significantly inhibited by 2 and 4 mg/mL XML. Over 2 mg/mL, XML inhibited the proliferation of H9C2 cells displaying moderate cytotoxicity. Therefore, we chose 0.25, 0.5, and 1 mg/mL XML for the subsequent research. To confirm the protective effect of XML on DOX-induced cardiotoxicity, we first established an acute DOX-associated cell model with 5 *μ*mol/L DOX and used three different concentrations of XML to treat H9c2 cells. As oxidative stress is the primary cause of DOX-induced cardiotoxicity, we measured cell proliferation and superoxide dismutase. As shown in Figures 1(b) and 1(c), DOX induced significant H9c2 cardiotoxicity presenting as lower cell proliferation and superoxide dismutase level. XML, particularly high concentration (1 mg/mL), could increase cell proliferation and SOD activity, thus reversing DOX-induced cell damage. Given these results, XML indeed alleviates DOX-induced H9c2 cardiomyocyte oxidative stress damage.

### 3.2. Effect of XML on Lysosome and Autophagy Degradation in DOX-Induced H9c2 Cells

**Figure 2:**
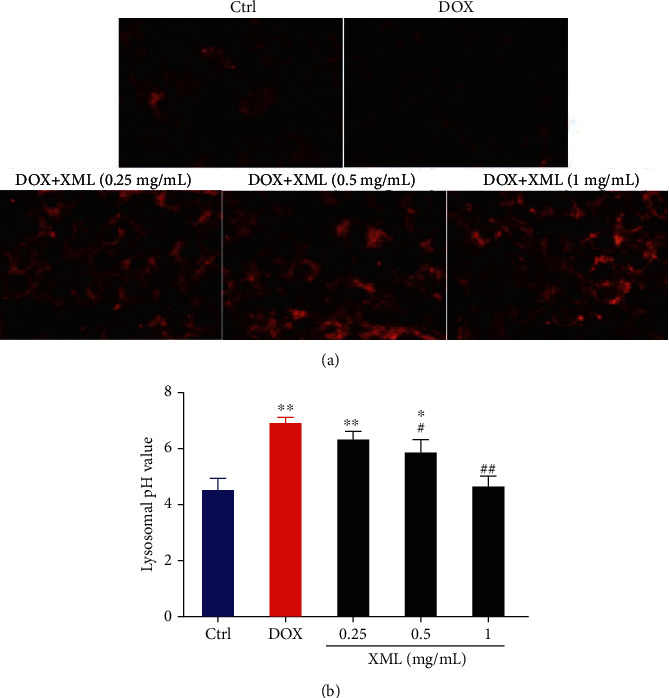
XML improves lysosomal function in DOX-induced H9c2 cells. (a) Lysosome was labeled by LysoTracker Red staining. (b) Lysosomal pH was determined by using fluorescein-dextran. H9c2 cells were treated with 5 *μ*mol/L DOX for 24 h only or preincubated with 0.25, 0.5, and 1 mg/mL XML for 48 h. Data are representative of at least three independent experiments (mean ± SD). Statistical significance was assigned as ^∗^*P* < 0.05 and ^∗∗^*P* < 0.01 compared with control and ^#^*P* < 0.05 compared with DOX. *P* values were determined using the independent one-way ANOVA and the Tukey-Kramer test. XML: Xinmailong treatment; DOX: doxorubicin.

**Figure 3:**
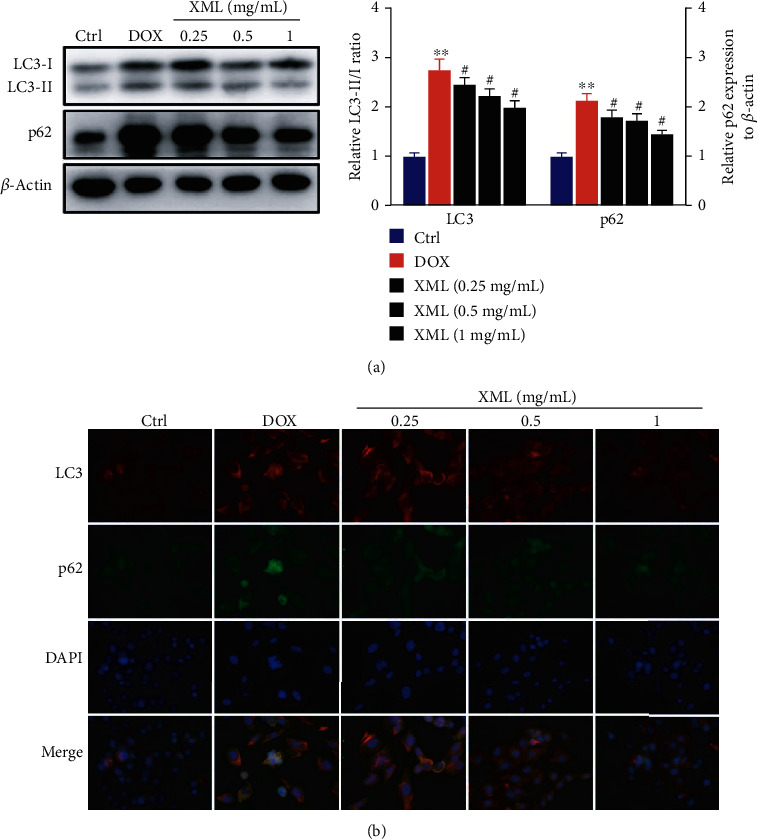
XML ameliorates autophagy flux block in DOX-induced H9c2 cells. (a) Western blotting of LC3 and p62 was represented with the LC3-II/I ratio and normalization to *β*-actin, respectively. (b) Representative immunofluorescence images of LC3 (red), p62 (green), and DAPI (blue). H9c2 cells were treated with 5 *μ*mol/L DOX for 24 h only or preincubated with 0.25, 0.5, and 1 mg/mL XML for 48 h. Data are representative of at least three independent experiments (mean ± SD). Statistical significance was assigned as ^∗^*P* < 0.05 and ^∗∗^*P* < 0.01 compared with control and ^#^*P* < 0.05 compared with DOX. *P* values were determined using the independent one-way ANOVA and the Tukey-Kramer test. XML: Xinmailong treatment; DOX: doxorubicin.

Previous studies revealed that DOX blocked cardiomyocyte autophagic flux and increased the degree of oxidative stress [22]. And the block mainly occurs in the autolysosome degradation stage, which could be caused by lysosomal dysfunction. First, we used LysoTracker Red staining to label lysosomes and detected the lysosomal pH value. As shown in Figure 2, DOX significantly decreased LysoTracker Red puncta and elevated the lysosomal pH. XML could increase LysoTracker Red puncta and reduce the lysosomal pH, and the high XML has the best effect. Meanwhile, we measured the autophagy flux related to protein LC3 and p62 expression, and the data showed that the levels of LC3-II/I and p62 were increased after DOX treatment compared with those in the control group, whereas XML treatment attenuated the accumulation of LC3-II/I and p62 (Figure 3(a)). The same phenomenon was observed by immunofluorescence (Figure 3(b)). These results demonstrated that XML may improve the lysosomal function and ameliorate autophagy flux block in DOX-treated H9c2 cells.

### 3.3. Effect of XML on HO-1 Expression in DOX-Induced H9c2 Cardiomyocytes

**Figure 4:**
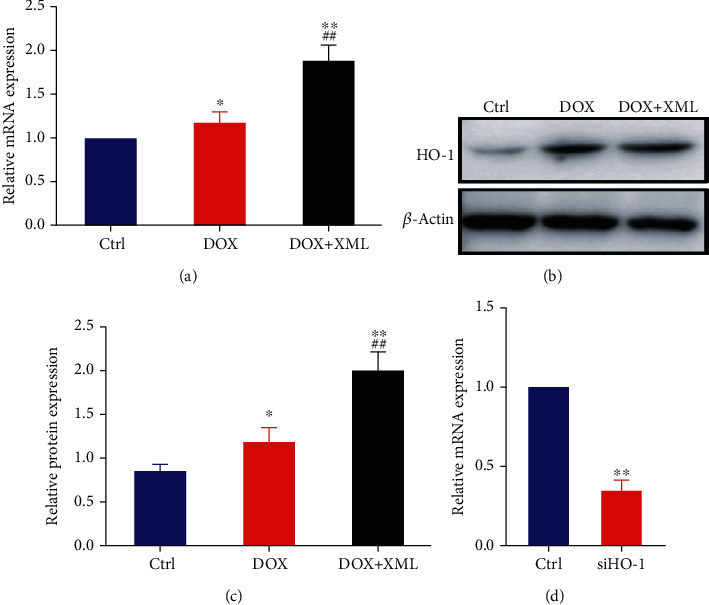
HO-1 expression was elevated in DOX-induced H9c2 cells treated with XML. (a) Real-time PCR of HO-1 was represented with normalization to GAPDH and *β*-actin. (b, c) Western blotting of HO-1 was represented with normalization to *β*-actin. H9c2 cells were treated with 5 *μ*mol/L DOX only or preincubated with 1 mg/mL XML for 48 h. (d) Real-time PCR of HO-1 was represented with normalization to GAPDH. Data are representative of at least three independent experiments (mean ± SD). Statistical significance was assigned as ^∗^*P* < 0.05 and ^∗∗^*P* < 0.01 compared with control and ^##^*P* < 0.01 compared with DOX. *P* values were determined using the independent one-way ANOVA and the Tukey-Kramer test. XML: Xinmailong treatment; DOX: doxorubicin.

Previous studies have demonstrated that HO-1 could protect against DOX-induced oxidative stress and maintain cellular homeostasis in several organs, including the heart [23]. To evaluate the potential role of HO-1 in XML treatment, we investigated the expression level of HO-1 in H9c2 cells treated with 1 mg/mL XML. As shown in Figures 4(a)–4(c), DOX treatment alone could increase HO-1 mRNA and protein levels compared with that of the control group and HO-1 expression was further increased when XML was added. These results showed that the elevated HO-1 serves as a feedback mechanism to suppress DOX-induced oxidative stress and XML supplementation effectively upregulated HO-1 expression.

### 3.4. HO-1 Regulates the Effect of XML on Oxidative Stress in DOX-Induced H9c2 Cells

**Figure 5:**
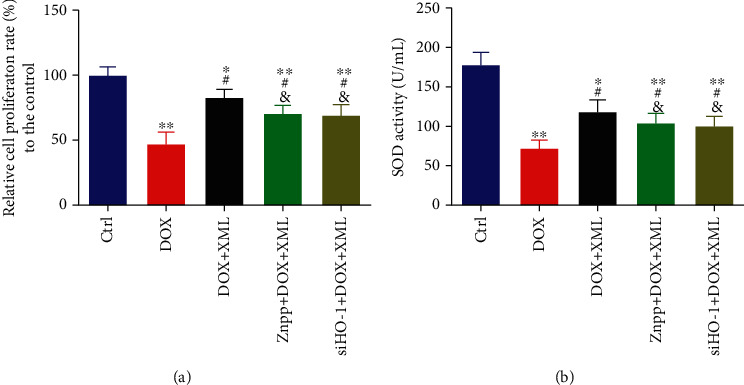
Downregulation of HO-1 abrogates the effect of XML on cell proliferation and SOD activity in DOX-induced H9c2 cells. (a) Cell proliferation was assessed by CCK-8. (b) Cell oxidative stress was reflected by SOD activity. H9c2 cells were treated with 5 *μ*mol/L DOX for 24 h only or preincubated with 1 mg/mL XML for 48 h. Data are representative of at least three independent experiments (mean ± SD). Statistical significance was assigned as ^∗^*P* < 0.05 and ^∗∗^*P* < 0.01 compared with DOX and ^#^*P* < 0.05 compared with DOX+XML. *P* values were determined using the independent one-way ANOVA and the Tukey-Kramer test. XML: Xinmailong treatment; DOX: doxorubicin; CCK8: Cell Counting Kit-8; SOD: superoxide dismutase.

To further confirm that XML attenuated DOX-induced cardiomyocyte oxidative stress damage through HO-1, the HO-1 inhibitor Znpp or HO-1 siRNA was utilized in this study. The confirmation of silencing is presented in Figure 4(d). As shown in Figure 5, coincubation with the HO-1 inhibitor Znpp or transfection with HO-1 siRNA abrogated the protective effects of XML on cell proliferation and superoxide dismutase activity. These results demonstrated that XML performed its anti-DOX cardiomyocyte protective role via HO-1.

### 3.5. HO-1 Regulates the Effect of XML on Lysosome and Autophagy Degradation in DOX-Induced H9c2 Cells

**Figure 6:**
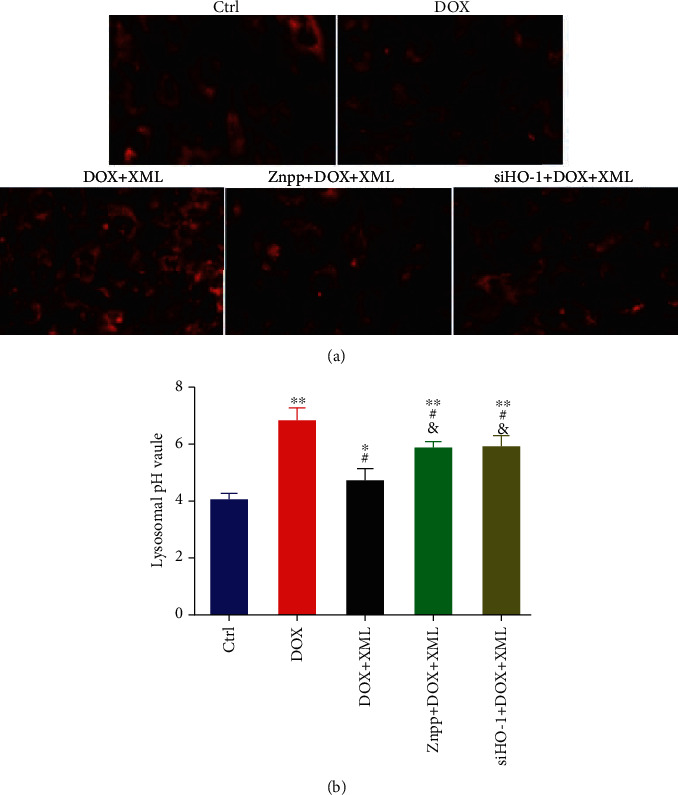
Downregulation of HO-1 abrogates the lysosome functions of XML in DOX-induced H9c2 cells. (a) Lysosome was labeled by LysoTracker Red staining. (b) Lysosomal pH was determined by using fluorescein-dextran. H9c2 cells were treated with 5 *μ*mol/L DOX for 24 h only or preincubated with 1 mg/mL XML for 48 h. Data are representative of at least three independent experiments (mean ± SD). Statistical significance was assigned as ^∗^*P* < 0.05 and ^∗∗^*P* < 0.01 compared with DOX and ^#^*P* < 0.05 compared with DOX+XML. *P* values were determined using the independent one-way ANOVA and the Tukey-Kramer test. XML: Xinmailong treatment; DOX: doxorubicin.

**Figure 7:**
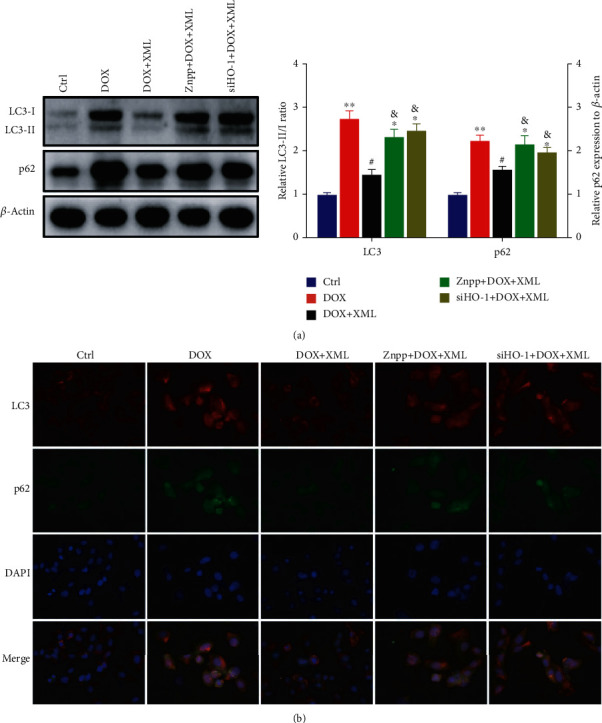
Downregulation of HO-1 abrogates the autophagy functions of XML in DOX-induced H9c2 cells. (a) Western blotting of LC3 and p62 was represented with the LC3-II/I ratio and normalization to *β*-actin, respectively. (b) Representative immunofluorescence images of LC3 (red), p62 (green), and DAPI (blue). H9c2 cells were treated with 5 *μ*mol/L DOX for 24 h only or preincubated with 1 mg/mL XML for 48 h. Data are representative of at least three independent experiments (mean ± SD). Statistical significance was assigned as ^∗^*P* < 0.05 and ^∗∗^*P* < 0.01 compared with DOX and ^#^*P* < 0.05 compared with DOX+XML. *P* values were determined using the two-tailed Student's *t*-test. XML: Xinmailong treatment; DOX: doxorubicin.

To reveal the underlying mechanism by which XML protected cardiomyocytes against DOX-induced injury, we detected lysosomal function and autophagy flux after inhibiting or silencing HO-1. As shown in Figure 6, coincubation with Znpp or transfection with HO-1 siRNA abolished the increase in LysoTracker Red puncta and the decrease in lysosomal pH caused by XML. Meanwhile, the XML-reduced LC3-II/I and p62 expressions were elevated by Znpp or HO-1 siRNA (Figure 7). The findings testified that silenced HO-1 could weaken the effect of XML on lysosome and autophagy degradation in DOX-induced H9c2 cells.

## 4. Discussion

Several studies indicated that XML could be used for therapy in patients with chronic heart failure [10, 24]. As a compound bioactive drug extracted from American cockroaches, the pharmacological effects of XML include promoting myocardial cell Ca^2+^ influx, reducing pulmonary artery pressure and capillary pressure, increasing coronary blood flow, and inhibiting oxygen free radical-mediated myocardial damage [25]. A component analysis study has identified 29 compounds in XML by liquid chromatography and gas chromatography mass spectrometry (HPLC-GC/MS), including polyols (38.5%), organic acids (18.8%), a variety of alkaloids (6.55%), and other ingredients [12]; furthermore, XML also has a protective effect on DOX-induced cardiotoxicity [12]. Despite extensive researches on DOX-induced cardiotoxicity, underlying molecular mechanisms are not completely clarified. Oxidative stress is one of the dominant theories about DOX-induced cardiotoxicity [26]; however, its pathophysiological process has remained to have some questions of debate. Antioxidant enzymes such as superoxide dismutase (SOD) play a pivotal role in lessening oxidative stress [27], while excessive oxidative stress inhibits cell proliferation and facilitates cell death [28]. Our results demonstrated that XML, particularly high concentration, could alleviate DOX-induced H9c2 cardiomyocyte oxidative stress damage.

To adapt to excessive oxidative stress, cells in the body initiate a series of regulatory response pathways, such as activating antioxidant enzymes. ROS produced during oxidative stress can induce autophagy through various mechanisms [29]. Autophagy is a cell self-protective mechanism for adapting to an adverse condition and maintaining intracellular homeostasis [30]. Impaired autophagy flux might promote cell damage by aggravating oxidative stress [31]. Moreover, oxidative stress damage could also induce autophagy [32], which potentially initiates a vicious cycle and aggravates cell damage. Several studies have reported that DOX could induce autophagy and block the degradation process of autophagy flux [33, 34], and our results confirmed this phenomenon as well. Li et al. reported [12] that XML mitigated epirubicin-induced cardiotoxicity via inhibiting autophagy, and they only determined the autophagy-related protein Beclin1 and ATG7 protein expression but did not monitor autophagy flux. Based on our results, we considered that XML treatment ameliorated the autophagy flux block.

Lysosomes play an essential role in the formation and degradation of autolysosomes [35]. In subsequent experiments, we further probed the effect of XML on lysosomal function in DOX-treated H9c2. We discovered that XML may improve the lysosomal function. At this time, the relationship remains unclear between lysosomes and oxidative stress. It has been reported that lysosomal dysfunction could promote oxidative stress [36], and the production of oxidative stress might be owing to defective degradation of autophagy flux or damaged mitochondria in autolysosomes [34].

HO-1, the inducible isoform of heme oxygenase (HO), was found to be an antioxidant and cytoprotective molecule [37]. It was transcriptionally regulated by NRF2 and plays a pivotal role in doxorubicin-induced oxidative stress [38]. Whether the effect of XML on alleviating oxidative stress is related to HO-1 has not been reported; therefore, our study showed that DOX slightly upregulated the expression of HO-1 and XML supplementation significantly elevated HO-1 expression. DOX-induced HO-1 upregulation could be considered a feedback mechanism to suppress DOX-induced oxidative stress, and HO-1 might be a target of XML to play its role in antioxidative stress damage.

To confirm our hypothesis, we further repeated the experiment via the HO-1 inhibitor Znpp or siRNA of HO-1. We discovered that inhibition of HO-1 partly abrogated the protective effects of XML on cell proliferation and oxidative stress in DOX-induced H9c2 cells, indicating that the effect of XML on alleviating oxidative stress is partially dependent on HO-1. In the present study, we have demonstrated for the first time that XML performed its anti-DOX cardiomyocyte protective role through HO-1. Meanwhile, inhibition of HO-1 weakened the effect of XML on improving lysosomal function and ameliorating autophagy flux block in DOX-induced H9c2 cells. XML indeed regulates lysosome and autophagy through HO-1. However, inhibition of HO-1 did not completely reverse the effects of XML in DOX-induced cardiotoxicity, and XML might be just partially dependent on HO-1. Other mechanisms need to be further studied in the future.

## 5. Conclusion

**Figure 8:**
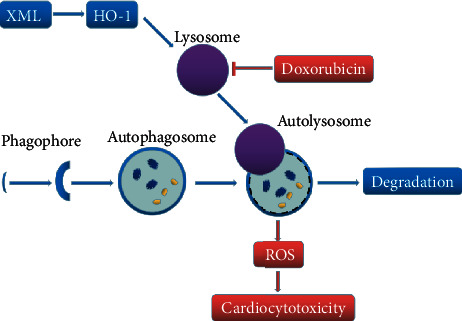
Graphical diagram representing the protective mechanism of XML against DOX-induced cardiotoxicity in H9c2 cells. Doxorubicin inhibits lysosomal function and blocks cardiomyocyte autophagy flux. Accumulation of autolysosomes increases ROS production and cell damage. XML rescues lysosomal function by increasing HO-1, then promotes autophagy flux and decreases the accumulation of autolysosomes, and finally reduces ROS and cell oxidative stress damage.

In conclusion, XML has protective effects against DOX-induced cardiotoxicity in H9c2 cells. XML improves DOX-induced cell proliferation, oxidative stress, and lysosome and autophagy dysregulation, which were partially mediated through HO-1 (Figure 8). These findings provided a theoretical basis and molecular mechanism for the implication of XML in DOX-induced cardiotoxicity.

## Data Availability

The data used to support the findings of this study are available from the corresponding author upon request.
